# Ethyl 2-amino-1-(4-fluoro­phen­yl)-5-oxo-4,5-di­hydro-1*H*-pyrrole-3-carboxyl­ate: crystal structure and Hirshfeld surface analysis

**DOI:** 10.1107/S2056989017011628

**Published:** 2017-08-11

**Authors:** U. H. Patel, Chintan Jotaniya, D. A. Shah, Bhavesh Socha

**Affiliations:** a Department of Physics, V.V. Nagar, Anand, Gujarat, India; b 103, X-Ray Lab, Department of Physics, V.V. Nagar, Anand, Gujarat, India; c Organic Synthesis Laboratory, M. G. Science Institute, Ahmedabad, Gujarat, India

**Keywords:** crystal structure, halogen-substituted pyrrole derivative, X-ray crystallography, Hirshfeld surface analysis, hydrogen bonding

## Abstract

In the title mol­ecule, C_12_H_13_FN_2_O_3_, the central pyrrole ring makes a dihedral angle of 9.2 (3)° with the eth­oxy carbonyl moiety whereas the fluoro­phenyl ring is rotated by 67.6 (2)° from the pyrrole ring. Supra­molecular aggregation is due to off-centric π–π stacking inter­actions involving screw-related pairs of mol­ecules, which are further connected by N—H⋯O and C—H⋯O inter­actions, forming a sinusoidal pattern along the [001] direction on the *bc* plane. Three-dimensional Hirshfeld surface analysis and two-dimensional fingerprint plots confirm the contributions of these inter­actions.

## Abstract 2

In the title mol­ecule, the central pyrrole ring makes dihedral angles of 9.2 (3) and 67.6 (2)°, respectively, with the eth­oxy carbonyl moiety and the fluoro­phenyl ring. Supra­molecular aggregation is due to off-centric π–π stacking inter­actions involving screw-related pairs of mol­ecules, which are further connected by N—H⋯O and C—H⋯O inter­actions, forming a sinusoidal pattern

## Chemical context   

Pyrrole, an electron-rich five-membered unsaturated ring, and its derivatives are widely used as inter­mediates in the synthesis of organic compounds, medicines, pharmaceuticals, agrochemicals, perfumes *etc*. Its derivatives possess a broad spectrum of biological activities. Substitution by a halogen (Cl, Br, F, I) is known to increase the activities of drug mol­ecules and this group of mol­ecules inter­act with receptors *via* halogen bonding. Organofluorine compounds display a variety of pharmacological and agro-chemical properties. Specific halogen-bonding inter­actions are responsible for the supra­molecular architecture in halogen-substituted heterocycles. Bearing in mind the importance of pyrrole and the role of halogens, we have synthesized a series of halogen-substituted pyrrole derivatives. Bromo and meth­oxy derivatives of the title mol­ecule have been reported earlier (Patel *et al.*, 2012 ▸, 2013 ▸). As a continuation of these studies, the title mol­ecule, with fluorine as one of the substituents, was synthesized and characterized crystallographically and by Hirshfeld surface analysis.
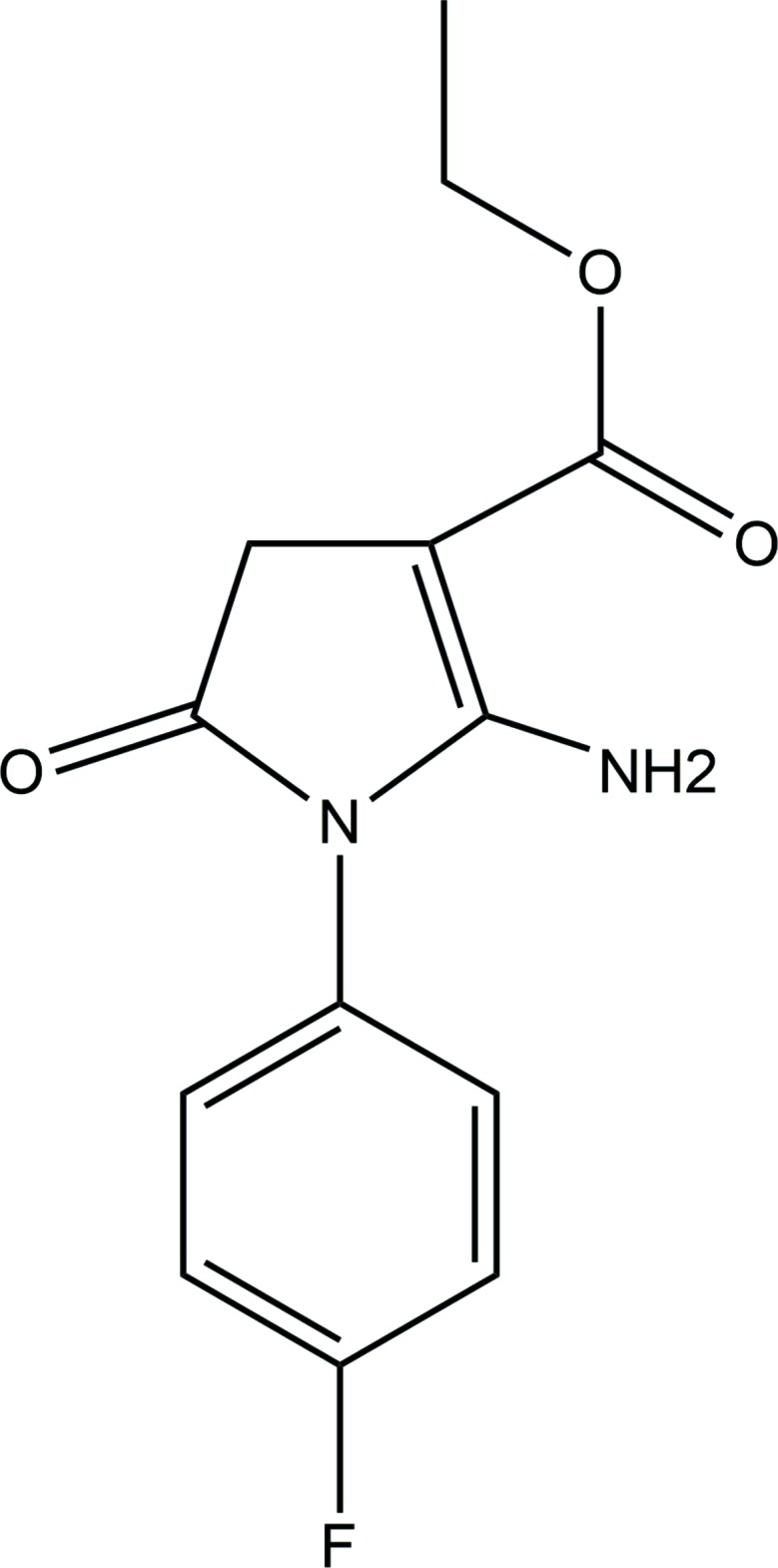


## Structural commentary   

**Figure 1:**
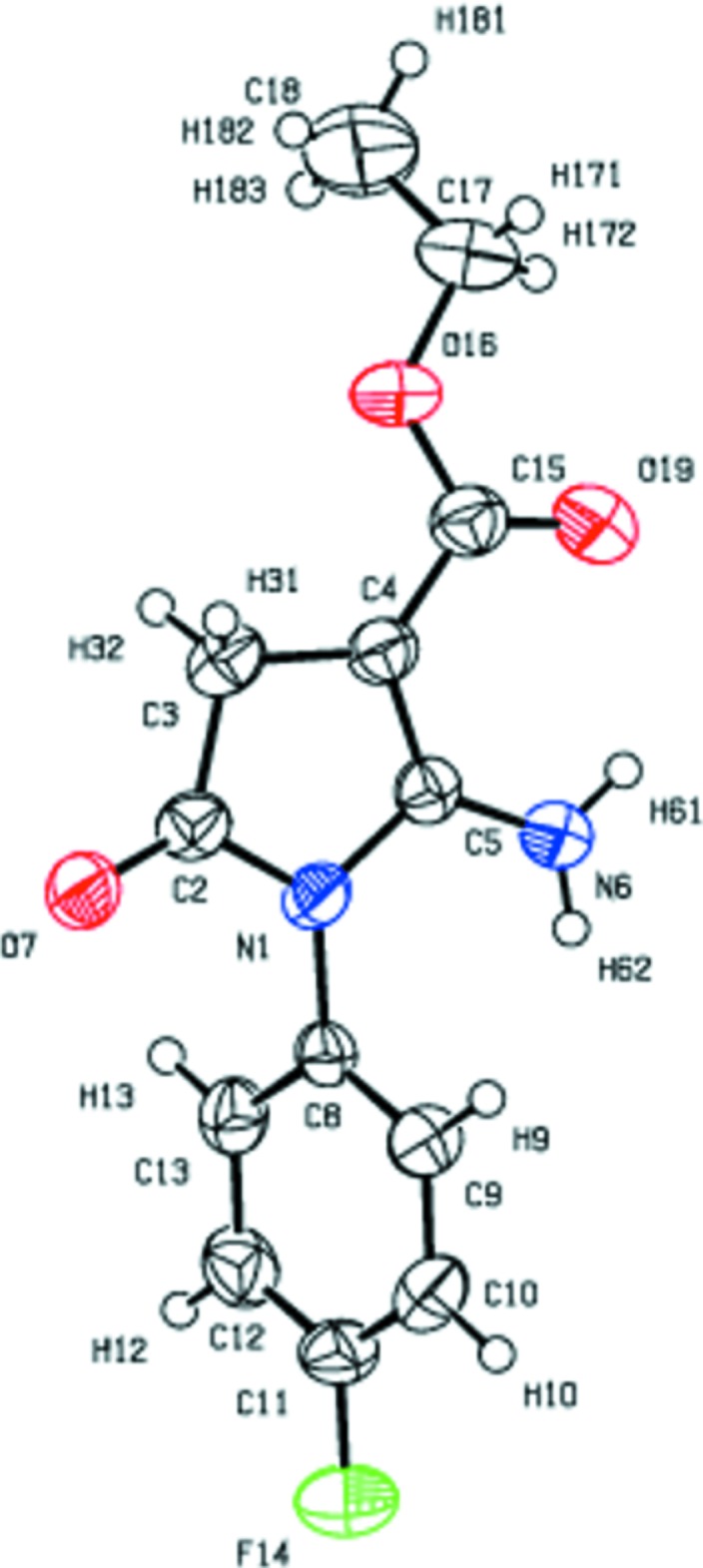
*ORTEP* view of the title mol­ecule with the atom-labelling scheme and displacement ellipsoids drawn at the 50% probability level.

In the title compound, Fig. 1 ▸, the F atom is displaced by 0.014 (3) Å from the phenyl ring, facilitating it in to take part in a number of inter­molecular inter­actions. The heterocyclic five-membered pyrrole ring is essentially planar with a maximum displacement of 0.022 (4) Å for atom C3 from its mean plane. The fluoro­phenyl ring forms a dihedral angle of 67.6 (2)° whereas the mean plane of eth­oxy carbonyl tail is inclined at 9.2 (3)° to the central pyrrole ring. The terminal eth­oxy carbonyl chain adopts a zigzag extended conformation, as is usually observed in analogous derivatives, with the carbonyl oxygen atom O19 on the same side as the methyl carbon atom C17 [C17—O16—C15—O19 = 5.0 (7)°] and the eth­oxy carbon atom C18 in a *trans* [C15—O16—C17—C18 = 144.6 (5)°] conformation with respect to the pyrrole ring. Bond lengths in the phenyl ring vary from 1.365 (6) to 1.385 (6) Å and the endocyclic angle varies from 118.0 (4) to 122.9 (4)° with an average value of 120.4 (4)°, which coincides exactly with the theoretical value 120° for *sp* ^2^ hybridization.

The intra­molecular N6—H61⋯O19 hydrogen bond involving the carbonyl oxygen atom O19 leads to the formation of a pseudo-six-membered ring with an *S*(6) graph-set motif.

## Supra­molecular features   

**Figure 2:**
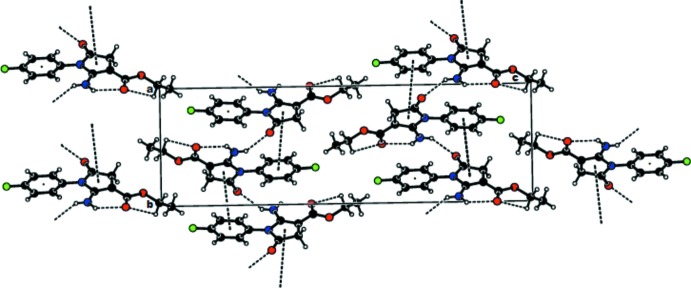
View of the packing showing π–π stacking inter­actions and N—H⋯O and C—H⋯O hydrogen bonds (dashed lines) in the *bc* plane.

In the crystal, two pairs of screw-related mol­ecules are held together by off-centric π–π stacking inter­actions involving the pyrrole ring and the phenyl ring of a screw-related mol­ecule (−*x*, 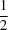 + *y*, 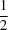 − *z*) [centroid–centroid distance = 4.179 (2) Å, slippage = 2.036 Å, dihedral angle between planes = 5.9 (2)°], forming chains along [010]. The structure contains infinite zigzag chains of screw-related mol­ecules, forming a sinusoidal patterns along [001] on the *bc* plane as shown in Fig. 2 ▸.

The molecular packing features N—H⋯O interactions, which lead to the formation of chains alon [001], and π–π stacking interactions, which link the molecules along [010]. In addition, C—H1⋯O inter­actions stack the molecules along [100] (Fig. 2 ▸, Table 1 ▸).

## Analysis of the Hirshfeld Surfaces   

**Figure 3:**
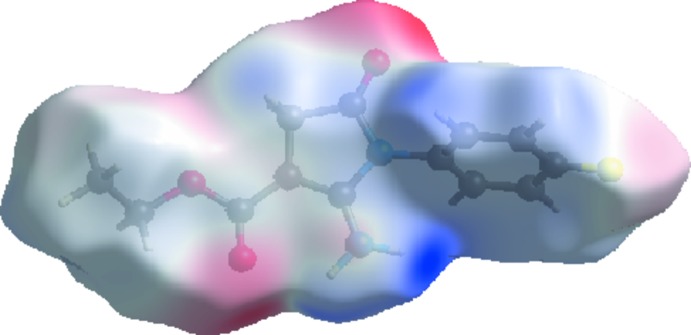
View of the Hirshfeld surface mapped over the calculated electrostatic potential for the title compound. The red and blue regions represent negative and positive electrostatic potentials, respectively.

*Crystal Explorer 3.1* (Wolff *et al.*, 2012 ▸) was used to generate Hirshfeld surfaces mapped over *d*_norm_, *d*_e_ and electrostatic potential for the title compound. The electrostatic potentials were calculated using *TONTO* (Spackman *et al.*, 2008 ▸; Jayatilaka *et al.*, 2005 ▸) as integrated in *Crystal Explorer* and are mapped on Hirshfeld surfaces using the STO-3G basis set at the Hartree–Fock level of theory over a range ±0.10 au as shown in Fig. 3 ▸. The positive electrostatic potential (blue region) over the surface indicates a hydrogen-bond donor, whereas the hydrogen-bond acceptors are represented by negative electrostatic potential (red region). The contact distances *d*_i_ and *d*_e_ from the Hirshfeld surface to the nearest atom inside and outside, respectively, enables the analysis of the inter­molecular inter­actions through the mapping of *d*_norm_.

**Figure 4:**
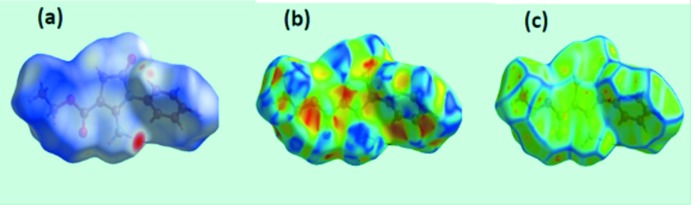
View of the Hirshfeld surface mapped over (*a*) *d*_norm_, (*b*) shape-index and (*c*) curvedness.

**Figure 5:**
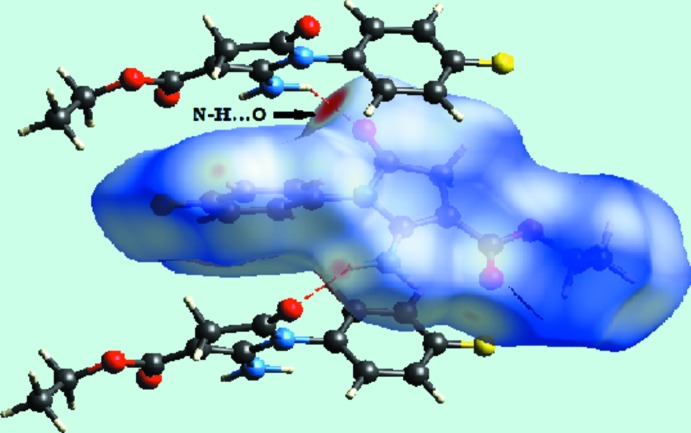
*d*_norm_ mapped on the Hirshfeld surface for visualizing the N—H⋯O inter­molecular inter­actions of the title compound. Red dotted lines represent hydrogen bonds.

**Figure 6:**
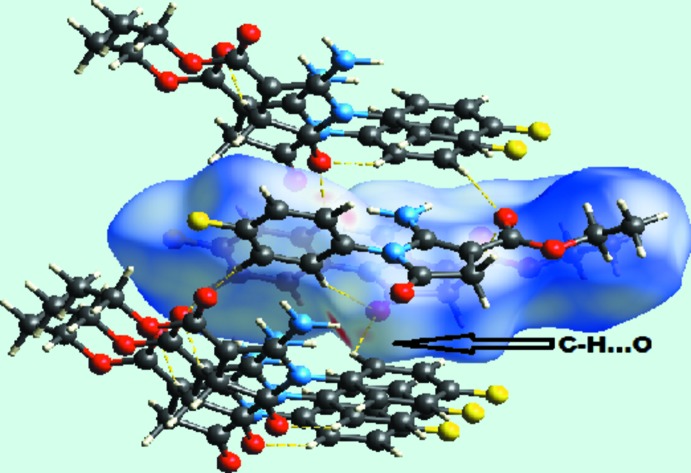
*d*_norm_ mapped on the Hirshfeld surface for visualizing the C—H⋯O inter­molecular inter­actions (yellow dotted lines) of the title compound.

A view of the Hirshfeld surface mapped over *d*_norm_, shape-index and curvedness for the title compound are shown in Fig. 4 ▸. Hirshfeld surfaces marked with red regions in *d*_norm_ near atoms O7, O19, N6, H62 and H10 reveal the active participation of the respective atoms in inter­molecular inter­actions. The occurrence of N—H⋯O and C—H⋯O inter­actions is confirmed by analysis of the Hirshfeld surface. N6—H62⋯O19 inter­actions are shown on the Hirshfeld surface marked with bright-red dotted lines in Fig. 5 ▸. Yellow dotted lines mapped on the *d*_norm_ Hirshfeld surface in Fig. 6 ▸ reveal the presence of C13—H13⋯O7 and C17—H172⋯O19 inter­actions.

**Figure 7:**
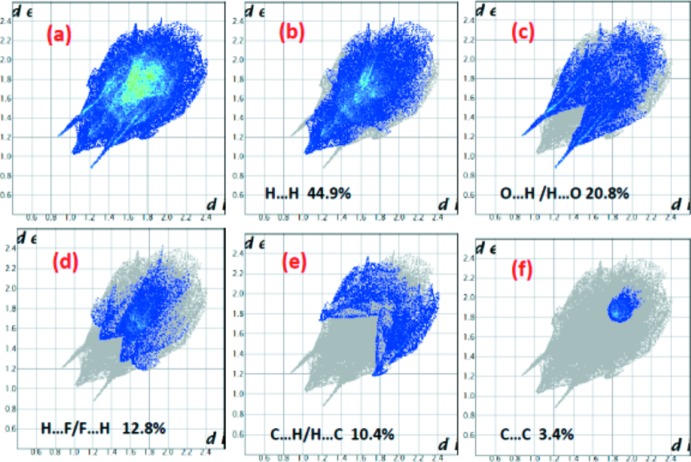
The two-dimensional fingerprint plots for the title compound, showing contributions from different contacts, (*a*) all, (*b*) H⋯H, (*c*) O⋯H/H⋯O, (*d*) H⋯F/F⋯H, (*e*) C⋯H/H⋯C and (*f*) C⋯C, respectively.

The two-dimensional fingerprint plots (Rohl *et al.*, 2008 ▸) for the title mol­ecule are shown in Fig. 7 ▸. The inter atomic H⋯H contacts appear as scattered points over the larger part of the plot along with one distinct spike with the highest contribution within the Hirshfeld surface of 44.9% (Fig. 7 ▸ *b*), followed by 20.8% for O⋯H/H⋯O contacts, which appear as pairs of adjacent spikes having almost same length. The contributions of H⋯F/F⋯H and C⋯H/H⋯C contacts are 12.8 and 10.4%, respectively. The contribution of C⋯C contacts, *i.e.* 3.0%, shows the π–π stacking inter­actions in the compound have a relatively smaller contribution. Apart from these, C⋯O/O⋯C, C⋯N/N⋯C, O⋯F/F⋯O, O⋯N/N⋯O and C⋯F/F⋯C contacts are found, as summarized in Table 2 ▸.

## Database survey   

Two analogous structures, 2-amino-1(4-bromo­phen­yl)-5-oxo-4,5-di­hydro-1*H*-pyrrole-3-carb­oxy­lic acid ethyl ester (Patel *et al.*, 2012 ▸) and 2-amino-1-(4-meth­oxy­phen­yl)-5-oxo-4,5-di­hydro-1*H*-pyrrole-3-carb­oxy­lic acid ethyl ester (Patel *et al.*, 2013 ▸), in which the fluoro­phenyl ring of the title compound is replaced by a bromo or meth­oxy­phenyl ring, are reported in the Cambridge Structural Database (Groom *et al.*, 2016 ▸).

## Synthesis and crystallization   

In a 50 ml flat-bottom flask, a mixture of dry toluene (15 ml), potassium hydroxide (0.012 mol, 0.672 g) and 18-crown-6 (0.0005 mol, 0.132 g) were prepared. Ethyl cyano­acetate (0.006 mol, 0.6787 g) was then added to this stirred mixture, followed by the portionwise addition of *N*-(4-fluoro­phen­yl)-2-chloro­acetamide (0.005 mol, 1.2425 g) after 5 min. The stirring was continued until the chloro­acetamide derivative had been consumed (20 min), monitored TLC (hexa­ne:ethyl acetate 7:3). On completion of the reaction, water (25 ml) was added to the reaction mixture and stirring continued for a further 5 min. This was then taken into a separating funnel and the aqueous phase was neutralized with glacial acetic acid (pH = 7). The phases were separated and the aqueous phase extracted with toluene (10 ml). The combined organic layers were dried over magnesium sulfate and the toluene removed *in vacuo* to obtain a solid product. The crude product was crystallized from ethanol to obtain 1.42 g (87% yield) of 2-amino-1-(4-fluoro­phen­yl)-oxo-4,5-di­hydro-1*H*-pyrrole-3-carb­oxy­lic acid ethyl ester, m.p. 783.24 K. It is more or less soluble in different solvents such as benzene, ethanol, DMF, DMSO, CH_2_CL_2_, CHCl_3_, ethyl acetate but diffraction quality crystal could be grown by the slow evaporation method at room temperature from ethyl acetate only after repeated trials.

## Refinement details   

Crystal data, data collection and structure refinement details are summarized in Table 3 ▸. Carbon-bound H atoms were placed in their calculated positions (C—H = 0.93–0.97 Å) and are included in the refinement in the riding-model approximation, with *U*_iso_(H) set to 1.2*U*_eq_(C).

## Supplementary Material

Crystal structure: contains datablock(s) global, I. DOI: 10.1107/S2056989017011628/ds2246sup1.cif

Structure factors: contains datablock(s) I. DOI: 10.1107/S2056989017011628/ds2246Isup2.hkl

CCDC reference: 1555656

Additional supporting information:  crystallographic information; 3D view; checkCIF report

## Figures and Tables

**Table 1:** Hydrogen-bond geometry (Å, °)

*D*—H⋯*A*	*D*—H	H⋯*A*	*D*⋯*A*	*D*—H⋯*A*
N6—H61⋯O19	0.86	2.2400	2.806 (4)	123
N6—H62⋯O7^i^	0.86	2.2100	2.970 (4)	147
C13—H13⋯O7^ii^	0.93	2.6000	3.320 (5)	135

Symmetry codes: (i) 
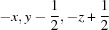
; (ii) 
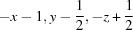
.

**Table 2:** Summary of various contacts and their percentage contributions to the Hirshfeld surface

Type of contact	Contribution
H⋯H	44.9
O⋯H/H⋯O	20.8
H⋯F/F⋯H	12.8
C⋯H/H⋯C	10.4
C⋯C	3.4
C⋯O/O⋯C	3.0
C⋯N/N⋯C	1.8
O⋯F/F⋯O	1.0
O⋯N/N⋯O	0.6
C⋯F/F⋯C	0.5

**Table 3:** Experimental details

Crystal data	
Chemical formula	C_13_H_13_FN_2_O_3_
*M*_r_	264.25
Crystal system, space group	Orthorhombic, *P*2_1_2_1_2_1_
Temperature (K)	273
*a*, *b*, *c* (Å)	5.5357 (16), 8.548 (2), 27.026 (7)
*V* (Å^3^)	1278.9 (6)
*Z*	4
Radiation type	Mo *K*α
μ (mm^−1^)	0.11
Crystal size (mm)	0.7 × 0.3 × 0.2

Data collection	
Diffractometer	Bruker SMART APEX CCD
Absorption correction	Multi-scan (*SADABS*; Bruker, 2007 ▸)
*T*_min_, *T*_max_	0.962, 0.979
No. of measured, independent and observed [*I* > 2Σ(*I*)] reflections	7696, 2975, 2322
*R*_int_	0.032

Refinement	
*R*[*F* ^2^ > 2σ(*F* ^2^)], *wR*(*F* ^2^), *S*	0.075, 0.148, 1.18
No. of reflections	2975
No. of parameters	173
H-atom treatment	H-atom parameters constrained
Δρ_max_, Δρ_min_ (e Å^−3^)	0.23, −0.24

Computer programs: *APEX2* and *SAINT* (Bruker, 2007 ▸), *SHELXS97* (Sheldrick, 2008 ▸), *SHELXL2013* (Sheldrick, 2015 ▸), *ORTEP-3 for Windows* (Farrugia, 2012 ▸) and *publCIF* (Westrip, 2010 ▸).

## supplementary crystallographic information

### Crystal data

**Table:**

C_13_H_13_FN_2_O_3_	*D*_x_ = 1.372 Mg m^−^^3^
*M**_r_* = 264.25	Melting point: 783.39 K
Orthorhombic, *P*2_1_2_1_2_1_	Mo *K*α radiation, λ = 0.71073 Å
Hall symbol: P 2ac 2ab	Cell parameters from 7696 reflections
*a* = 5.5357 (16) Å	θ = 1.5–28.2°
*b* = 8.548 (2) Å	µ = 0.11 mm^−^^1^
*c* = 27.026 (7) Å	*T* = 273 K
*V* = 1278.9 (6) Å^3^	Transparent, colourless
*Z* = 4	0.7 × 0.3 × 0.2 mm
*F*(000) = 552	

### Data collection

**Table:**

Bruker SMART APEX CCD diffractometer	2975 independent reflections
Radiation source: SEALED TUBE	2322 reflections with *I* > 2Σ(*I*)
Graphite monochromator	*R*_int_ = 0.032
ω–2θ scan	θ_max_ = 28.2°, θ_min_ = 1.5°
Absorption correction: multi-scan (SADABS; Bruker, 2007)	*h* = −7→6
*T*_min_ = 0.962, *T*_max_ = 0.979	*k* = −10→11
7696 measured reflections	*l* = −34→32

### Refinement

**Table:**

Refinement on *F*^2^	0 restraints
Least-squares matrix: full	Hydrogen site location: inferred from neighbouring sites
*R*[*F*^2^ > 2σ(*F*^2^)] = 0.075	H-atom parameters constrained
*wR*(*F*^2^) = 0.148	Weighting scheme based on measured s.u.'s
*S* = 1.18	(Δ/σ)_max_ = 0.006
2975 reflections	Δρ_max_ = 0.23 e Å^−^^3^
173 parameters	Δρ_min_ = −0.24 e Å^−^^3^

### Special details

**Table:**

Geometry. Bond distances, angles etc. have been calculated using the rounded fractional coordinates. All su's are estimated from the variances of the (full) variance-covariance matrix. The cell esds are taken into account in the estimation of distances, angles and torsion angles

### Fractional atomic coordinates and isotropic or equivalent isotropic displacement parameters (Å^2^)

**Table:**

	*x*	*y*	*z*	*U*_iso_*/*U*_eq_	
F14	0.0103 (6)	0.1908 (3)	0.41628 (9)	0.0777 (11)	
O7	−0.3466 (5)	0.3868 (3)	0.20473 (10)	0.0501 (9)	
O16	0.0782 (6)	0.1173 (4)	0.04855 (10)	0.0675 (11)	
O19	0.3634 (6)	0.0024 (4)	0.09592 (11)	0.0665 (11)	
N1	−0.0226 (5)	0.2174 (3)	0.21258 (11)	0.0380 (9)	
N6	0.3131 (6)	0.0484 (3)	0.19821 (11)	0.0449 (10)	
C2	−0.1947 (7)	0.3016 (4)	0.18607 (14)	0.0402 (11)	
C3	−0.1510 (8)	0.2676 (4)	0.13223 (14)	0.0457 (12)	
C4	0.0711 (7)	0.1663 (4)	0.13244 (13)	0.0403 (11)	
C5	0.1342 (6)	0.1388 (4)	0.18000 (13)	0.0351 (11)	
C8	−0.0114 (6)	0.2105 (4)	0.26584 (12)	0.0345 (11)	
C9	0.1786 (7)	0.2794 (4)	0.29063 (14)	0.0420 (12)	
C10	0.1843 (8)	0.2732 (5)	0.34181 (15)	0.0503 (12)	
C11	0.0017 (8)	0.1985 (5)	0.36617 (14)	0.0490 (14)	
C12	−0.1879 (8)	0.1282 (5)	0.34218 (15)	0.0527 (16)	
C13	−0.1941 (7)	0.1362 (4)	0.29129 (14)	0.0437 (12)	
C15	0.1869 (8)	0.0873 (5)	0.09193 (14)	0.0473 (12)	
C17	0.1672 (12)	0.0330 (8)	0.00528 (17)	0.094 (2)	
C18	−0.0234 (14)	−0.0020 (10)	−0.0268 (2)	0.134 (4)	
H9	0.30124	0.32939	0.27321	0.0506*	
H10	0.31042	0.31912	0.35929	0.0606*	
H12	−0.30851	0.07676	0.35975	0.0630*	
H13	−0.32196	0.09126	0.27406	0.0526*	
H31	−0.12308	0.36342	0.11386	0.0551*	
H32	−0.28713	0.21277	0.11777	0.0551*	
H61	0.40656	−0.00181	0.17837	0.0539*	
H62	0.33375	0.04110	0.22967	0.0539*	
H171	0.28599	0.09643	−0.01185	0.1126*	
H172	0.24516	−0.06313	0.01573	0.1126*	
H181	0.03741	−0.05787	−0.05497	0.2012*	
H182	−0.09846	0.09335	−0.03758	0.2012*	
H183	−0.14019	−0.06545	−0.00986	0.2012*	

### Atomic displacement parameters (Å^2^)

**Table:**

	*U*^11^	*U*^22^	*U*^33^	*U*^12^	*U*^13^	*U*^23^
F14	0.087 (2)	0.109 (2)	0.0371 (14)	0.006 (2)	−0.0004 (14)	0.0091 (13)
O7	0.0503 (16)	0.0487 (14)	0.0512 (16)	0.0128 (15)	−0.0068 (14)	−0.0092 (12)
O16	0.071 (2)	0.098 (2)	0.0334 (15)	0.011 (2)	−0.0043 (14)	−0.0106 (16)
O19	0.064 (2)	0.084 (2)	0.0516 (18)	0.017 (2)	0.0054 (15)	−0.0114 (16)
N1	0.0386 (17)	0.0400 (15)	0.0355 (17)	0.0025 (15)	−0.0067 (14)	0.0008 (13)
N6	0.0452 (19)	0.0525 (18)	0.0371 (17)	0.0057 (17)	0.0003 (15)	−0.0050 (14)
C2	0.046 (2)	0.0337 (18)	0.041 (2)	0.0002 (19)	−0.0057 (19)	−0.0048 (16)
C3	0.050 (2)	0.043 (2)	0.044 (2)	−0.001 (2)	−0.012 (2)	−0.0030 (17)
C4	0.042 (2)	0.0419 (19)	0.037 (2)	0.0004 (17)	−0.0040 (17)	−0.0021 (17)
C5	0.0337 (19)	0.0340 (17)	0.0377 (19)	−0.0045 (16)	−0.0009 (16)	−0.0005 (15)
C8	0.034 (2)	0.0346 (17)	0.0349 (19)	0.0072 (17)	0.0009 (16)	−0.0007 (14)
C9	0.042 (2)	0.043 (2)	0.041 (2)	−0.008 (2)	−0.0008 (18)	0.0053 (16)
C10	0.045 (2)	0.057 (2)	0.049 (2)	0.000 (2)	−0.012 (2)	−0.0007 (19)
C11	0.057 (3)	0.059 (2)	0.031 (2)	0.010 (2)	0.000 (2)	0.0080 (18)
C12	0.045 (2)	0.062 (3)	0.051 (3)	−0.001 (2)	0.011 (2)	0.011 (2)
C13	0.036 (2)	0.045 (2)	0.050 (2)	−0.0025 (19)	−0.0008 (18)	−0.0012 (18)
C15	0.048 (2)	0.052 (2)	0.042 (2)	−0.006 (2)	−0.0012 (19)	−0.0025 (18)
C17	0.091 (4)	0.146 (5)	0.045 (3)	0.017 (5)	0.002 (3)	−0.025 (3)
C18	0.108 (6)	0.205 (8)	0.090 (4)	0.009 (6)	−0.017 (4)	−0.078 (5)

### Geometric parameters (Å, º)

**Table:**

F14—C11	1.357 (5)	C9—C10	1.385 (6)
O7—C2	1.221 (5)	C10—C11	1.365 (6)
O16—C15	1.343 (5)	C11—C12	1.372 (6)
O16—C17	1.459 (6)	C12—C13	1.378 (6)
O19—C15	1.222 (6)	C17—C18	1.398 (9)
N1—C2	1.393 (5)	C3—H31	0.9700
N1—C5	1.407 (4)	C3—H32	0.9700
N1—C8	1.442 (4)	C9—H9	0.9300
N6—C5	1.349 (5)	C10—H10	0.9300
C2—C3	1.503 (5)	C12—H12	0.9300
C3—C4	1.504 (6)	C13—H13	0.9300
C4—C5	1.353 (5)	C17—H171	0.9700
C4—C15	1.437 (5)	C17—H172	0.9700
N6—H61	0.8600	C18—H181	0.9600
N6—H62	0.8600	C18—H182	0.9600
C8—C13	1.378 (5)	C18—H183	0.9600
C8—C9	1.379 (5)		
			
C15—O16—C17	117.0 (4)	O16—C15—C4	112.1 (4)
C2—N1—C5	110.3 (3)	O19—C15—C4	124.7 (4)
C2—N1—C8	124.4 (3)	O16—C17—C18	110.4 (5)
C5—N1—C8	125.4 (3)	C2—C3—H31	111.00
O7—C2—N1	124.5 (3)	C2—C3—H32	111.00
O7—C2—C3	128.7 (4)	C4—C3—H31	111.00
N1—C2—C3	106.7 (3)	C4—C3—H32	111.00
C2—C3—C4	103.8 (3)	H31—C3—H32	109.00
C3—C4—C5	108.4 (3)	C8—C9—H9	120.00
C3—C4—C15	129.2 (3)	C10—C9—H9	120.00
C5—C4—C15	121.8 (3)	C9—C10—H10	121.00
N1—C5—N6	119.9 (3)	C11—C10—H10	121.00
N1—C5—C4	110.6 (3)	C11—C12—H12	121.00
N6—C5—C4	129.5 (3)	C13—C12—H12	121.00
C5—N6—H62	120.00	C8—C13—H13	120.00
H61—N6—H62	120.00	C12—C13—H13	120.00
C5—N6—H61	120.00	O16—C17—H171	110.00
C9—C8—C13	120.9 (3)	O16—C17—H172	110.00
N1—C8—C13	119.1 (3)	C18—C17—H171	110.00
N1—C8—C9	120.0 (3)	C18—C17—H172	110.00
C8—C9—C10	119.1 (4)	H171—C17—H172	108.00
C9—C10—C11	118.9 (4)	C17—C18—H181	109.00
F14—C11—C10	118.6 (4)	C17—C18—H182	109.00
F14—C11—C12	118.5 (4)	C17—C18—H183	109.00
C10—C11—C12	122.9 (4)	H181—C18—H182	109.00
C11—C12—C13	118.0 (4)	H181—C18—H183	109.00
C8—C13—C12	120.2 (4)	H182—C18—H183	109.00
O16—C15—O19	123.3 (4)		
			
C17—O16—C15—O19	5.0 (7)	C3—C4—C5—N6	176.4 (3)
C17—O16—C15—C4	−174.9 (4)	C15—C4—C5—N1	−173.6 (3)
C15—O16—C17—C18	144.6 (5)	C15—C4—C5—N6	4.7 (6)
C5—N1—C2—O7	−176.3 (3)	C3—C4—C15—O16	2.6 (6)
C8—N1—C2—O7	4.7 (5)	C3—C4—C5—N1	−1.9 (4)
C5—N1—C2—C3	2.9 (4)	C3—C4—C15—O19	−177.3 (4)
C5—N1—C8—C9	69.1 (4)	C5—C4—C15—O16	172.5 (4)
C2—N1—C8—C13	67.1 (4)	C5—C4—C15—O19	−7.4 (7)
C5—N1—C8—C13	−111.7 (4)	N1—C8—C9—C10	179.2 (3)
C2—N1—C5—N6	−179.2 (3)	C13—C8—C9—C10	0.0 (5)
C8—N1—C5—N6	−0.2 (5)	N1—C8—C13—C12	−179.9 (3)
C2—N1—C5—C4	−0.7 (4)	C9—C8—C13—C12	−0.7 (5)
C8—N1—C5—C4	178.3 (3)	C8—C9—C10—C11	0.2 (6)
C2—N1—C8—C9	−112.1 (4)	C9—C10—C11—C12	0.3 (7)
C8—N1—C2—C3	−176.1 (3)	C9—C10—C11—F14	179.0 (4)
O7—C2—C3—C4	175.4 (4)	F14—C11—C12—C13	−179.7 (4)
N1—C2—C3—C4	−3.8 (4)	C10—C11—C12—C13	−1.0 (7)
C2—C3—C4—C5	3.5 (4)	C11—C12—C13—C8	1.2 (6)
C2—C3—C4—C15	174.4 (4)		

### Hydrogen-bond geometry (Å, º)

**Table:**

*D*—H···*A*	*D*—H	H···*A*	*D*···*A*	*D*—H···*A*
N6—H61···O19	0.86	2.2400	2.806 (4)	123
N6—H62···O7^i^	0.86	2.2100	2.970 (4)	147
C13—H13···O7^ii^	0.93	2.6000	3.320 (5)	135
C17—H172···O19	0.97	2.3300	2.692 (6)	101

Symmetry codes: (i) −*x*, *y*−1/2, −*z*+1/2; (ii) −*x*−1, *y*−1/2, −*z*+1/2.
